# Diagnosis of Obstructive Sleep Apnea Using Feature Selection, Classification Methods, and Data Grouping Based Age, Sex, and Race

**DOI:** 10.3390/diagnostics13142417

**Published:** 2023-07-20

**Authors:** Alaa Sheta, Thaer Thaher, Salim R. Surani, Hamza Turabieh, Malik Braik, Jingwei Too, Noor Abu-El-Rub, Majdi Mafarjah, Hamouda Chantar, Shyam Subramanian

**Affiliations:** 1 Computer Science Department, Southern Connecticut State University, New Haven, CT 06514, USA; shetaa1@southernct.edu; 2 Department of Computer Systems Engineering, Arab American University, Jenin P.O. Box 240, Palestine; 3 Department of Pulmonary, Critical Care & Sleep Medicine, Texas A&M University, College Station, TX 77843, USA; surani@tamu.edu; 4 Health Management and Informatics Department, School of Medicine, University of Missouri, Columbia, MO 65212, USA; hit8zp@health.missouri.edu; 5 Department of Computer Science, Al-Balqa Applied University, Salt 19117, Jordan; mbraik@bau.edu.jo; 6 Faculty of Electrical Engineering, Universiti Teknikal Malaysia Melaka, Hang Tuah Jaya, Durian Tunggal 76100, Melaka, Malaysia; jamesjames868@gmail.com; 7 Center of Medical Informatics and Enterprise Analytics, University of Kansas Medical Center, Kansas City, KS 66160, USA; nabuelrub@kumc.edu; 8 Department of Computer Science, Birzeit University, Birzeit P.O. Box 14, Palestine; mmafarja@birzeit.edu; 9 Faculty of Information Technology, Sebha University, Sebha 18758, Libya; ham.chantar@sebhau.edu.ly; 10 Pulmonary, Critical Care & Sleep Medicine, Sutter Health, Tracy, CA 95376, USA; subramanian@sutterhealth.org

**Keywords:** obstructive sleep apnea, grouping, feature selection, machine learning

## Abstract

Obstructive sleep apnea (OSA) is a prevalent sleep disorder that affects approximately 3–7% of males and 2–5% of females. In the United States alone, 50–70 million adults suffer from various sleep disorders. OSA is characterized by recurrent episodes of breathing cessation during sleep, thereby leading to adverse effects such as daytime sleepiness, cognitive impairment, and reduced concentration. It also contributes to an increased risk of cardiovascular conditions and adversely impacts patient overall quality of life. As a result, numerous researchers have focused on developing automated detection models to identify OSA and address these limitations effectively and accurately. This study explored the potential benefits of utilizing machine learning methods based on demographic information for diagnosing the OSA syndrome. We gathered a comprehensive dataset from the Torr Sleep Center in Corpus Christi, Texas, USA. The dataset comprises 31 features, including demographic characteristics such as race, age, sex, BMI, Epworth score, M. Friedman tongue position, snoring, and more. We devised a novel process encompassing pre-processing, data grouping, feature selection, and machine learning classification methods to achieve the research objectives. The classification methods employed in this study encompass decision tree (DT), naive Bayes (NB), k-nearest neighbor (kNN), support vector machine (SVM), linear discriminant analysis (LDA), logistic regression (LR), and subspace discriminant (Ensemble) classifiers. Through rigorous experimentation, the results indicated the superior performance of the optimized kNN and SVM classifiers for accurately classifying sleep apnea. Moreover, significant enhancements in model accuracy were observed when utilizing the selected demographic variables and employing data grouping techniques. For instance, the accuracy percentage demonstrated an approximate improvement of 4.5%, 5%, and 10% with the feature selection approach when applied to the grouped data of Caucasians, females, and individuals aged 50 or below, respectively. Furthermore, a comparison with prior studies confirmed that effective data grouping and proper feature selection yielded superior performance in OSA detection when combined with an appropriate classification method. Overall, the findings of this research highlight the importance of leveraging demographic information, employing proper feature selection techniques, and utilizing optimized classification models for accurate and efficient OSA diagnosis.

## 1. Introduction

Obstructive sleep apnea (OSA) is a severe respiratory disorder that was first introduced in 1837 by Charles Dickens [1]. The foremost common symptoms of OSA are loud snoring, dry mouth upon awakening, morning headaches, and concentration difficulties [2,3]. There are over 100 million patients who suffer from sleep apnea, and it can affect both adults and children [4,5,6]. Moreover, it is estimated that nearly 22 million Americans suffer from a type of apnea that varies from moderate to severe [7]. Typically, the apnea–hypopnea index (AHI) is used to measure the severity of the apnea. For example, with nearly 326 million people living in the USA, it’s reported that 10% of the US population have mild OSA with AHI scores larger than 5, 3.5% have moderate OSA with AHI scores larger than 15, and 4% have severe OSA syndrome (i.e., apnea/hypopnea) [7].

The publication titled “Hidden health crisis costing America billions” by the American Academy of Sleep Medicine (AASM) presents a new analysis that sheds light on the considerable economic consequences of undiagnosed OSA [8]. Neglecting sleep apnea significantly raises the likelihood of expensive health complications such as hypertension, heart disease, diabetes, and depression [9]. By examining 506 patients diagnosed with OSA, the study showcases the potential improvements in their quality of life following treatment, including enhanced sleep quality, increased productivity, and a notable 40% reduction in workplace absences. A substantial 78% of patients regarded their treatment as a significant investment. Frost & Sullivan, a leading market research firm, has estimated the annual economic burden of undiagnosed sleep apnea among adults in the United States to be approximately $149.6 billion. This staggering amount encompasses $86.9 billion in lost productivity, $26.2 billion in motor vehicle accidents, and $6.5 billion in workplace accidents. Sleep apnea can be categorized into three distinct types:

- Obstructive sleep apnea (OSA): The most common type of apnea is known as obstructive sleep apnea (OSA), which is identified by two primary characteristics. The first is a continuous reduction in airflow of at least 30% for a duration of 10 seconds, which is accompanied by a minimum oxygen desaturation of 4%. The second is a decrease in airflow of at least 50% for 10 seconds, coupled with a 3% reduction in oxygen saturation [10].
- Central sleep apnea (CSA): CSA occurs when the brain fails to send appropriate signals to the muscles responsible for breathing. Unlike OSA, which stems from mechanical issues, CSA arises due to impaired communication between the brain and muscles [11,12].
- Mixed sleep apnea (MSA): MSA, also known as complex sleep apnea, represents a combination of obstructive and central sleep apnea disorders, thus presenting a more complex pattern of symptoms and characteristics.

Detecting OSA using an electrocardiogram (ECG) is an expensive process that is inaccessible to a large number of the world’s population. The attributes of the ECG signal differ in the case of awake and sleep intervals [13]. Hence, using a combined signal of awake and sleep stages reduces the overall reliability of the detection process. Several researchers recommend examining the ECG signal based on minutes [14]. In general, to detect OSA, the signal length should be at least 10 seconds in length. The diagnosis of OSA from ECG signals using various machine learning methods is a commonly used approach in the literature. For example, artificial neural networks (ANN) and convolutional neural networks (CNN) were introduced to detect and classify OSA. Wang et al. [15] used the CNN model to detect OSA based on ECG signals. The authors extracted a set of features from each signal and then trained a three-layered CNN model. The obtained results showed an acceptable performance and the ability to apply the proposed method over wearable devices.

Erdenebayar et al. [16] provided an automated detection method for OSA using a single-lead ECG and a CNN. The CNN model proposed in their study was meticulously constructed, featuring six convolution layers that were carefully optimized. These layers incorporated activation functions, pooling operations, and dropout layers. The research findings demonstrated that the proposed CNN model exhibited remarkable accuracy in detecting OSA solely by analyzing a single-lead ECG signal. Faust et al. [17] introduced the use of a long short-term memory (LSTM) neural network to detect sleep apnea based on RR intervals signal. Their results showed the ability of the LSTM network to detect sleep apnea with an accuracy equal to 99.80%. Schwartz et al. [18] employed several machine learning methods to detect four types of abbreviated digital sleep questionnaires (DSQs). The authors showed the ability of machine learning in detecting sleep disturbances with high accuracy. Lakhan et al. [19] proposed a dramatic involvement of a deep learning approach to detect multiple sleep apnea–hypopnea syndrome (SAHS). Two types of classifications were employed in their paper: binary classification with three cutoff indices (i.e., AHI = 5, 15, and 30 events/hour) and multiclass classification (i.e., no SAHS, mild SAHS, moderate SAHS, and severe SAHS). The obtained results for the binary classification showed that an AHI with 30 events/hour outperformed other cutoffs with an accuracy of 92.69%. For multiclass classification problems, the obtained accuracy was 63.70%. Banluesombatkul et al. [20] employed a novel deep learning method to detect OSA (i.e., normal and severe patients). The proposed method used three different deep learning methods: (i) one-dimensional CNN (1-D CNN) for feature extraction; (ii) deep recurrent neural networks (DRNNs) with an LSTM network for temporal information extraction; and (iii) fully connected neural networks (DNNs) for feature encoding. The proposed method showed acceptable results compared to the literature.

There have been several efforts to identify the relation between the snoring sound and OSA in the literature. In general, loud snoring is one of the indicators of OSA, and it is commonly thought that the frequency and amplitude of snoring are associated with the severity of the OSA [21]. Alshaer et al. [22] employed an acoustic analysis of breath sounds to detect OSA. The previous research suggests that OSA can be detected using snoring attributes. However, clinicians should pay attention to the possibility of missing an OSA diagnosis for patients with minimal snoring. Kang et al. [23] applied linear predict coding (LPC) and Mel-frequency cepstral coefficient (MFCC) features to detect OSA based on the amplitude of the snoring signal. The proposed method was able to classify three different events, namely, snoring, apnea, and silence, from sleep recordings with accuracies of 90.65%, 90.99%, and 90.30%, respectively.

Feature extraction and feature selection are the most commonly used techniques for data dimensionality reduction. Several papers have been published that highlight the importance of feature selection in OSA detection. Various features are extracted from the ECG signals; then, feature selection is used to reduce the number of extracted features and to determine the most valuable features related to OSA. In the stage of feature extraction, a set of features is extracted from the time series data, which aims to reveal the hidden information within the ECG signal. However, a feature set may contain redundant and irrelevant information, and feature selection is adopted to resolve this issue. A feature selection algorithm can help find the nearly optimal combination of features. Although feature selection is an expensive method, it can produce better classification performance, and high accuracy is significantly important in OSA detection. There are different classification methods that are used to select important features, such as support vector machine (SVM) networks, k-nearest neighbor (kNN) algorithms, artificial neural networks (ANN), linear discriminant analysis (LDA), and logistic regression (LR).

Many researchers have used demographic data to identify OSA. Sheta et al. [24,25] applied LR and ANN models to detect OSA based on demographic data. A real dataset was used that consists of several demographic features (i.e., weight, height, hip, waist, BMI, neck size, age, snoring, the modified Friedman (MF) score, the Epworth sleepiness scale, sex, and daytime sleepiness). The obtained results suggested that the proposed method could detect OSA with an acceptable accuracy. Surani et al. [26] applied the AdaBoost method as a machine learning classifier to detect OSA based on demographic data. The obtained results were promising. Surani et al. [27] applied a wrapper feature selection method based on binary particle swarm optimization (BPSO) with an ANN to detect OSA. The obtained results illustrated that the use of BPSO with an ANN can detect OSA with high accuracy. Haberfeld et al. [28] proposed a mobile application called Sleep Apnea Screener (SAS) to detect OSA based on demographic data. The authors used nine demographic features (i.e., height, weight, waist, hip, BMI, age, neck, M. Friedman, Epworth, snoring, gender, and daytime sleepiness). The application had two machine learning methods: LR and SVM. Moreover, the authors studied the performance of each classifier based on gender. The reported results showed that the proposed application can help patients detect OSA easily compared to an overnight test for OSA diagnosis.

There are many screening approaches for OSA, including tools such as the Berlin Questionnaire, the STOP-BANG Questionnaire, Epworth Sleepiness Scale (ESS), clinical assessment, and population-specific screening tools [29]. These approaches aim to identify individuals at a higher risk of OSA based on symptoms, risk factors, and questionnaire responses. Positive screening results prompt further evaluation using diagnostic tests such as polysomnography (PSG) or home sleep apnea testing (HSAT). Screening helps prioritize resources and directs individuals toward comprehensive sleep assessments. Subramanian et al. [30] introduced a novel screening approach known as the NAMES, which employs statistical methods to identify OSA. The NAMES assessment combines various factors, including neck circumference, airway classification, comorbidities, the Epworth scale, and snoring, to create a comprehensive evaluation that incorporates medical records, current symptoms, and physical examination findings. Experimental findings demonstrated the efficacy of the NAMES assessment in detecting OSA. Furthermore, the inclusion of BMI and gender in the assessment improved its screening capabilities.

This work proposes an efficient classification framework for the early detection of OSA. In specific, it is an extension of the NAMES work machine learning classification method and utilizes a metaheuristic-based feature selection scheme. The main contributions are summarized as follows:

1. The OSA data was grouped based on age, sex, and race variables for performance improvement. This type of grouping is novel and has never been presented in this area of research before.
2. Various types of the most well-known machine learning algorithms were assessed to determine the best-performing one for the OSA problem. These methods included twelve predefined (fixed) parameter classifiers and two optimized classifiers (using hyperparameter optimization).
3. A wrapper feature selection approach using particle swarm optimization (PSO) was employed to determine the most valuable features related to the OSA.
4. Experimental results from the actual data (collected from Torr Sleep Center, Texas, USA) confirmed that the proposed method improved the overall performance of the OSA prediction.

The rest of this paper is organized as follows: Section 2 presents the proposed method used in this work. Section 3 gives a brief description of the dataset used in the experiment. Section 5 discusses the experimental results and simulations. Finally, the conclusion and future work are presented in Section 8.

## 2. Proposed Diagnosis Process

**Figure 1:**
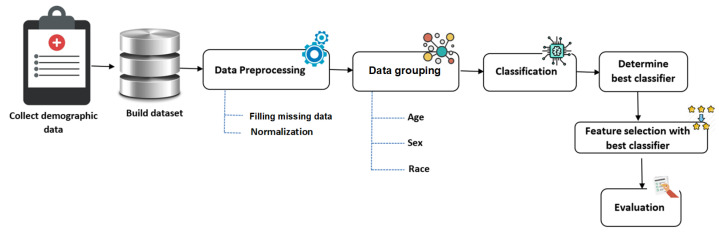
The proposed methodology. The figure illustrates the step-by-step process of the proposed methodology, which involved five steps: data collection, data preprocessing, data grouping, classification, feature selection, and evaluation.

The proposed OSA diagnosis process is illustrated in Figure 1. We suggest collecting data from patients who have undergone demographic, anthropometric measurements, and polysomnographic studies from a community-based sleep laboratory. An expert from the Torr Sleep Center (Corpus Christi, TX, USA) controlled the collection process for the polysomnography (PSG) evaluation of suspected OSA between 5 February 2007, and 21 April 2008. We processed the data to make the data more suitable for the analysis process. All missing data were handled, and a normalization technique was employed to transform the data into a standard scale. The next step was classification-based grouped data, where the classification model was implemented based on two types of learning methods—fixed parameter setting and adaptive parameter setting—through the training process. The benefit of using two kinds of learning methods is to learn more about the dataset and find the optimal parameter settings. After that, we applied a wrapper feature selection using the best performing classifier to identify the most valuable features related to OSA. This step can reveal useful information to physicians and doctors to understand the demographic characteristics of OSA patients. Finally, we used a set of evaluation criteria (i.e., accuracy, TPR, TNR, AUC, precision, F-score, and G-mean) to evaluate the performance of each classifier.

## 3. Sleep Apnea Dataset

**Figure 2:**
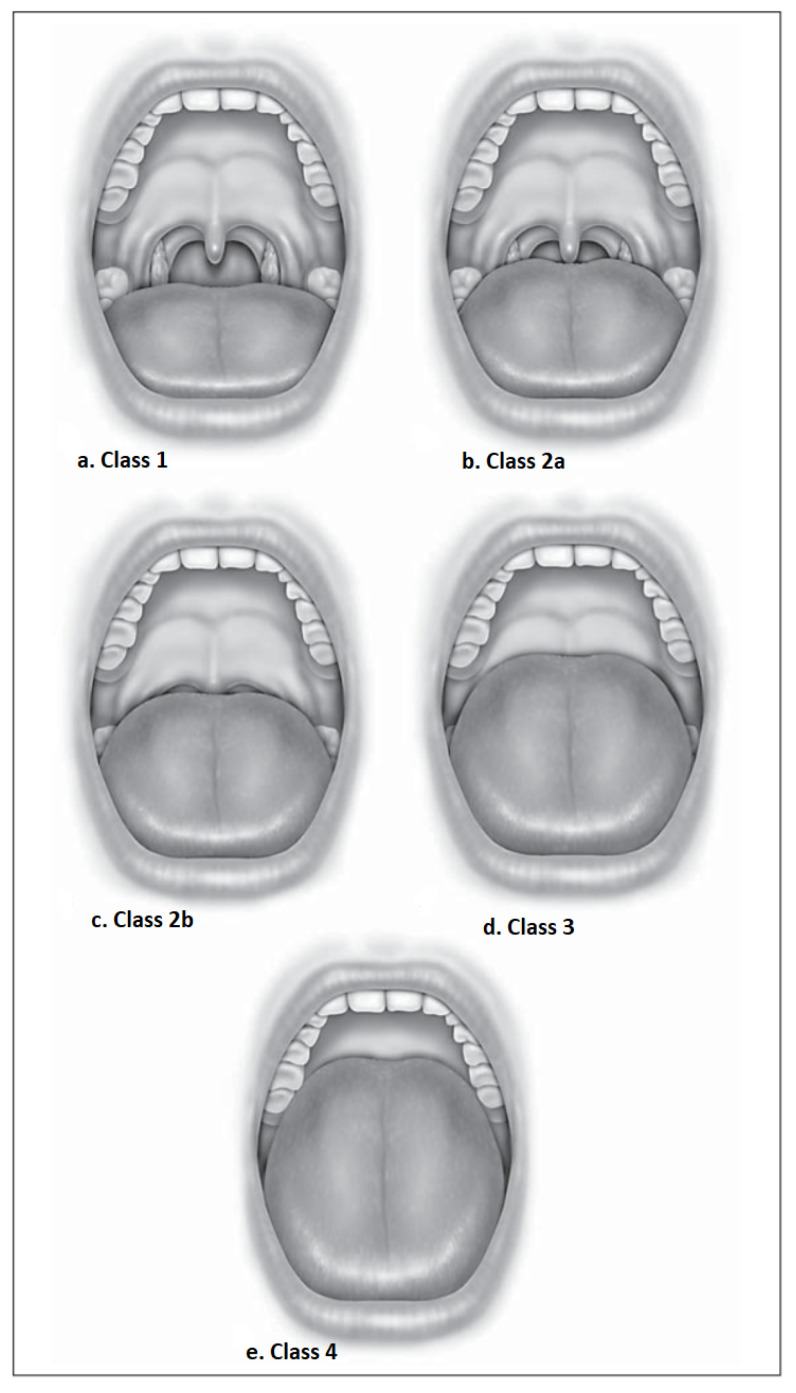
Friedman tongue position (FTP): (**a**) Class 1 visualizes the uvula and tonsils/pillar. (**b**) Class 2a visualizes most of the uvula but not the tonsils/pillar. (**c**) Class 2b visualizes the entire soft palate to the uvular base. (**d**) Class 3 shows some of the soft palates with the distal end absent. (**e**) Class 4 visualizes only the hard palate [31].

The initial dataset employed in this study encompasses 620 patients, comprising 366 males and 254 females. The age range for males spans from 19 to 88 years, while for females, it ranges from 20 to 96 years. Notably, the prevalence of snoring was 92.6% among males and 91.7% among females. Each patient underwent comprehensive full-night monitoring as part of the study. The dataset comprises 31 input features and a binary output, represented by either 0 or 1, thus indicating the presence or absence of obstructive sleep apnea (OSA) (see Table 1 for a detailed presentation of these features). Additionally, the study recorded each individual’s Friedman tongue position (FTP), which encompasses four distinct positions, as depicted in Figure 2. Additionally, the Epworth scale, which is used to assess sleepiness, was collected. The scale details are presented in Table 2. Notably, the dataset is imbalanced, with 357 patients identified as positive cases with OSA and 263 individuals identified without OSA. Table 3 provides a comprehensive overview of the dataset’s characteristics.

## 4. Data Preprocessing

In the data classification-process-based machine learning, data preprocessing is when the data gets encoded to transfer it to a state that the machine can quickly analyze. In this case, the features of the data were smoothly interpreted by the algorithm. Data preprocessing is a vital step in any machine learning process [32]. This process aims to reduce unexpected behavior through the learning process, thereby enhancing the machine learning algorithm’s performance [33,34]. A set of operations such as data cleaning, data transformation, and data reduction are usually involved in data preprocessing. Precisely, the main preprocessing steps used in this research are the following: fill the missing values, data grouping, normalization, and feature selection.

### 4.1. Missing Data

It is ubiquitous to have missing elements from either rows or columns in your dataset. A failure to collect accurate data can occur during the data collection process or be due to a particular adopted data validation rule. There are several methods to handle missing data. They include the following:

- If more than 50% of any rows or columns values are missing, we have to remove the whole row/columns, except where it is feasible to fill in the missing values.
- If only a rational percentage of values are missing, we can adopt simple interpolation methods to fill in those values. Interpolation methods include filling missing values with the mean, median, or mode value of the respective feature.

**Figure 3:**
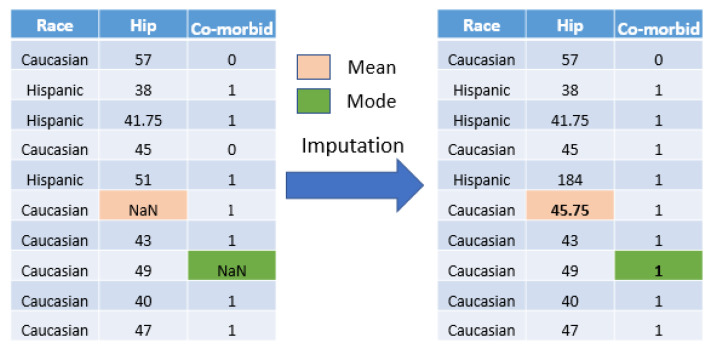
Imputation of Missing Values. NaN refers to a missing value. Mean is a statistical measure used for imputing missing numerical values by replacing them with the mean value of the available data. Mode is a statistical measure used for imputing missing categorical values by replacing them with the mode (most frequently occurring value) of the available data.

In this work, we applied a statistical imputation approach [35,36]. All missing values for each attribute were replaced with a statistical measure that was calculated from the remaining values for that attribute. The statistics used were the mean and mode for the numeric and nominal features, respectively. These methods were chosen because they are fast, easy to implement, prevent information loss, and work well with a small dataset. Figure 3 depicts an example of missing value imputation.

### 4.2. Data Normalization

Data normalization is the process of standardizing numerical attributes into a common scale [37,38]. This operation is strongly recommended for the machine learning process to avoid any bias towards dominant features. Min–max normalization was applied in this research to rescale every numerical feature value into a number within [0,1]. For every feature, every value *x* gets transformed into $x^{n}$ using formula given in Equation (1).

(1) $$x^{n}=\frac{x-min}{max-min},$$

where $x^{n}$ is the normalized value of *x*, and min and max represent the minimum and maximum value of the feature, respectively.

### 4.3. Role of Grouping in OSA Diagnosis

Recently, there have been many research efforts toward understanding the relationship between sex, age, and ethnicity in the diagnosis of sleep apnea.

Several research articles have explored the concept of data grouping. For example, in a study conducted by Mohsenin et al. [39], the authors examined the relationship between gender and the prevalence of hypertension in individuals with obstructive sleep apnea (OSA). The study, based on a large cohort of patients assessed at the Yale Center for Sleep Medicine, investigated how gender influences the likelihood of hypertension in OSA patients. The results revealed that hypertension rates increased with age and the severity of OSA, with obese men in the clinic-based population being at approximately twice the risk of hypertension compared to women. Similarly, another study by Freitas et al. [40] investigated the impact of gender on the diagnosis and treatment of OSA.

The study conducted by Ralls et al. [41] delved into the roles of gender, age, race/ethnicity, and residential socioeconomics in OSA syndromes. The research reviewed the existing literature and shed light on several intriguing findings. OSA was found to predominantly affect males, while women exhibited lower apnea–hypopnea index (AHI) values than men during specific sleep stages. Interestingly, women required lower levels of continuous positive airway pressure (CPAP) for treating OSAs of similar severities. The study also highlighted the impact of environmental factors, such as obesity, craniofacial structure, lower socioeconomic status, and residing in disadvantaged neighborhoods, on the prevalence and severity of OSA among different ethnic and racial groups.

In a research paper by Slaats et al. [42], an investigation was conducted to explore the relationship between ethnicity and OSA, which specifically focused on upper airway (UA) morphology, including Down syndrome. The findings of the study revealed that black African (bA) children exhibited a distinct upper airway morphology and were more prone to experiencing severe and persistent OSA compared to Caucasian children. This suggests that ethnicity plays a role in the susceptibility to OSA and highlights the importance of considering ethnic differences in diagnosing and managing the condition.

The Victoria Sleep Cohort study, as discussed in Irene et al. [43], investigated the gender-related impact of OSA on cardiovascular diseases. The study found consistent evidence linking OSA with cardiovascular risk, with a particular emphasis on men with OSA. The authors highlighted that the relationship between OSA and cardiovascular risk is influenced by gender, thereby indicating the need for tailored OSA treatment approaches for men and women. Additionally, Mohsenin et al. [44] conducted a study examining the effect of obesity on pharyngeal size separately for men and women, thus providing insights into the influence of obesity on the upper airway in OSA patients of different genders.

**Figure 4:**
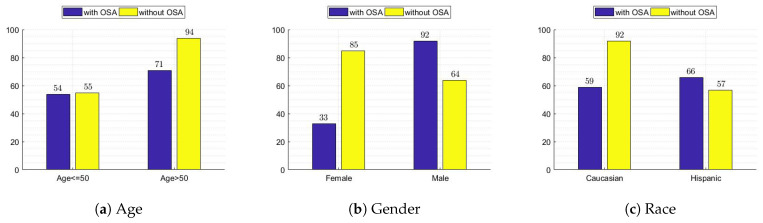
Bar charts comparing data samples with and without obstructive sleep apnea (OSA) grouped by age, gender, and race. The charts provide insights into the prevalence of OSA within different demographics.

One of the main objectives of this study is to investigate the detection of OSA before and after grouping data based on demographic variables such as age, gender, and race. Accordingly, the original data was grouped by ethnicity (Caucasian and Hispanic), gender (males and females), and age (age ≤ 50 or age > 50). Consequently, six datasets were investigated: Caucasian, Hispanic, females, males, age ≤ 50, and age > 50. In Table 3, we are showing the data-distribution-based grouping. In Figure 4, we show the distribution of apnea and no apnea with respect to age, gender, and race attributes for all datasets.

### 4.4. Wrapper Feature Selection

Feature selection (FS) plays a crucial role in data mining, wherein it serves as a preprocessing phase to identify and retain informative patterns/features while excluding irrelevant ones. This NP-hard optimization problem has significant implications in data classification, as selecting valuable features can enhance the classification accuracy and reduce computational costs [45,46].

FS methods can be categorized into two families based on the criteria used to evaluate the selected feature subset: these include filters and wrappers [46,47]. Filter FS techniques employ scoring matrices to assign weights to features, such as mutual information or chi-square tests. Features with weights below a threshold are then eliminated from the feature set. On the other hand, wrapper FS methods utilize classification algorithms such as SVM or linear discriminant analysis to assess the quality of the feature subsets generated by a search method [48,49].

Generally, wrapper FS approaches tend to yield higher classification accuracy by leveraging dependencies among features within a subset. In contrast, filter FS methods may overlook such dependencies. However, wrapper FS comes with a higher computational cost compared to filter FS [50].

Feature subset generation involves the search for a highly informative subset of features from a set of patterns. Various search strategies, such as heuristic, complete, and random, are employed for this purpose [51,52,53]. The complete search involves generating and examining all possible feature subsets in the search space to identify the most informative one. However, this approach becomes computationally infeasible for large datasets due to the exponential growth of subsets. For instance, if a dataset has 31 features, the complete search would generate $2^{31}$ subsets for evaluation. Random search, as the name implies, randomly explores the feature space to find subsequent feature subsets [54]. Although random search can, in some cases, generate all possible feature subsets similar to a complete search [45,55], it lacks a systematic search pattern.

In contrast, heuristic search is a different approach used to feature subset generation. It is characterized by iteratively improving the quality of the solution (i.e., a feature subset) based on a given heuristic function, thereby aiming to optimize a specific problem [56]. While heuristic search does not guarantee finding the best solution, it can often find good solutions within reasonable memory and time constraints. Several metaheuristic algorithms, such as particle swarm optimization (PSO) [57], ant colony optimization (ACO) [58], the firefly algorithm (FA) [59], ant lion optimization (ALO) [60], the whale optimization algorithm (WOA) [61], and the grey wolf optimizer (GWO) [62], have demonstrated their effectiveness in addressing feature subset selection problems. Examples of FS approaches can be found in [63,64,65,66,67,68,69].

This paper presents a wrapper feature selection approach based on particle swarm optimization (PSO) [70]. The main concept behind PSO is to simulate the collective behavior of bird flocking. The algorithm initializes a group of particles (solutions) that explore the search space in order to find the optimal solution for a given optimization problem. Each particle in the population adjusts its velocity and position based on the best solution found so far within the swarm. By considering the best particle, each individual particle updates its velocity and position according to specific rules, as outlined in Equations (2) and (3).

The PSO-based wrapper feature selection approach described in the paper utilizes this algorithm to search for an effective feature subset that improves the performance of the chosen optimization problem. For further details, please refer to [70,71].

(2) $$\begin{array}{ccc} v_{i}^{j}\left(m+1\right) & = & \omega_{1}v_{i}^{j}\left(m\right)+c_{1}r_{1}\left(pbest_{i}^{j}-x_{i}^{j}\left(m\right)\right)+c_{2}r_{2}\left(gbest_{i}^{j}-x_{i}^{j}\left(m\right)\right) \end{array}$$

(3) $$x_{i}^{j}\left(m+1\right)=x_{i}^{j}\left(m\right)+v_{i}^{j}\left(m+1\right),$$

where *m* denotes the current generation, $\omega_{1}$ is a parameter, named inertia weight, that is used for controlling the global search and local search tendencies. $v_{i}^{j}\left(m\right)$ denotes the current velocity at generation *m* for the *j*-th dimension of the *i*-th particle, and $x_{i}^{j}\left(m\right)$ denotes the current position of the *i*-th particle for the *j*-th dimension. Two uniformly distributed randomly assigned numbers between (0,1) are presented by $r_{1}$ and $r_{2}$, respectively, and $c_{1}$ and $c_{2}$ are known as acceleration coefficients. pbest is the optimal solution that the particle *i* has found so far. gbest refers to the best solution found within the population so far.

To adapt the original PSO algorithm for discrete or binary search space, a modified binary version was introduced by [57]. The primary step in this transformation is the utilization of a sigmoid (transfer) function, as shown in Equation (4), to convert the real-valued velocities into probability values ranging from 0 to 1. The objective is to adjust the particle’s position based on the probability defined by its velocity. This allows for the representation of binary or discrete variables within the PSO framework.

(4) $$S\left(v_{i}^{j}\left(m\right)\right)=\frac{1}{1+\exp^{-v_{i}^{j}\left(m\right)}},$$

where $v_{i}^{j}\left(m\right)$ refers to the velocity of particle *i* at iteration *m* in the *j*-th dimension. The updating process for the S-shape group is presented in Equation (5) for the next iteration m+1. After that, the position vectors can be updated based on the probability values of their velocities as follows:

(5) $$x_{i}^{j}\left(m+1\right)=\left\{\begin{array}{cc} 0 & \mathrm{If}\;rand<S(v_{i}^{j}\left(m+1\right))\\ 1 & \mathrm{If}\;rand\geq S(v_{i}^{j}\left(m+1\right)). \end{array}\right.$$

**Figure 5:**
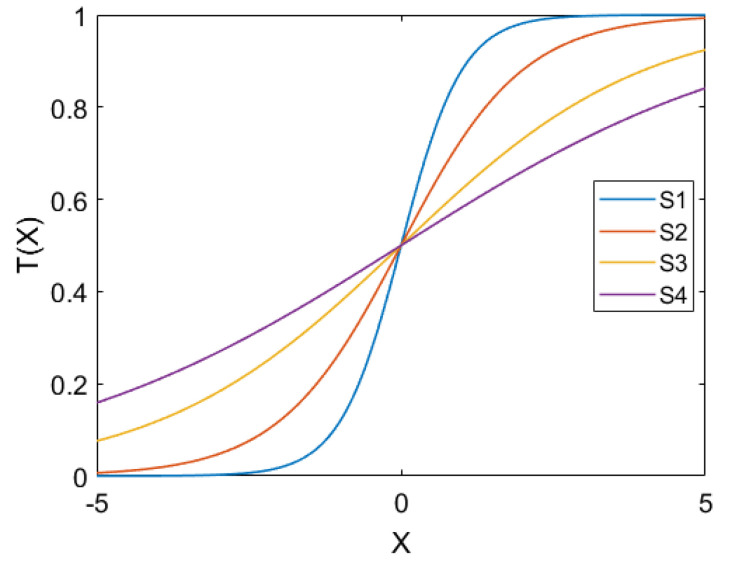
S-shaped TFs. The figure illustrates the curves of four distinct S-shaped transfer functions.

The basic version of the BPSO suffers from some drawbacks, such as trapping in local minima. Mirjalili and Lewis [71] proposed a modified version of the BPSO in which transfer functions for mapping continuous search the space into binary were employed. The aim of introducing these functions is to avoid the problem of local optima and to improve the convergence speed. In this work, we employed the S-shaped transfer functions proposed in [71] for converting the PSO into binary. We examined these functions with the PSO algorithm to choose the most appropriate one. Table 4 presents the utilized transfer functions, and Figure 5 shows the shapes of these transfer functions.

### 4.5. Formulation of Feature Selection Problem

An FS is typically treated as a binary optimization problem, where candidate solutions are represented as binary vectors. To address this, a binary optimizer such as binary particle swarm optimization (BPSO) can be utilized. This work proposes a wrapper FS method that combines the BPSO as the search strategy and a classifier (e.g., KNN) to evaluate the quality of the feature subsets generated by the BPSO. In the FS problem, a solution is encoded as a binary vector with a length equal to the total number of features in the dataset. Each element in the vector represents a feature, where a value of zero indicates the exclusion of the corresponding feature, and a value of one indicates its inclusion or selection.

The paper introduces four FS approaches based on different binary variants of PSO, with each utilizing a specific S-shaped transfer function to convert continuous values into binary ones. The FS is considered to be a multi-objective optimization problem, thereby aiming to achieve both high classification accuracy and a low number of features. These two objectives are formulated as contradictory objectives in Equation (6) [46,64].

(6) $$↓Fitness=\alpha \times er+\beta \times \frac{F}{N},$$

where er indicates the error rate of the utilized classification algorithm (e.g., KNN) over a subset of features produced by the BPSO optimizer. *F* is the number of selected features, and *N* denotes the number of all the features. α=0.99 and β=0.01 [72,73] are two controlling parameters to balance the importance of both objectives.

## 5. Experimental Setup

It is well-known that there is no universal machine learning algorithm that can be the best-performing for all problems (As suggested by the No Free Lunch (NFL) theorem [74]). This motivated our attempts to examine various fixed and adaptive classification algorithms to identify the most applicable one for OSA. In the experiment, various classification methods were tested. However, only those classifiers with better performances are reported. Correspondingly, we adopted the decision tree (DT), naive Bayes (NB), K-nearest neighbor (kNN), support vector machine (SVM), fine decision tree (FDT), coarse decision tree (CDT), linear discriminate analysis (LDA), logistic regression (LR), Gaussian naive Bayes (GNB), kernel naive Bayes (KNB), linear support vector machine (LSVM), medium Gaussian support vector machine (MGSVM), coarse Gaussian support vector machine (CGSVM), cosine k-nearest neighbor (CKNN), weighted K-nearest neighbor (WKNN), and subspace discriminant (Ensemble) classifiers for performance validation. The detailed parameter settings for these classification methods are presented in Table 5. Moreover, the kNN and SVM with hyperparameter optimization settings (see Table 6 and Table 7) were also employed in this work.

In this study, a K-fold cross-validation with K = 10 was employed for performance evaluation instead of a hold-out validation. K-fold cross-validation offers the advantage of estimating the generalization error by using different combinations of training and testing sets. This approach allows for comprehensive testing of the data. For assessing the performance of the machine learning models, multiple metrics were utilized, including the accuracy, true positive rate (TPR), true negative rate (TNR), area under the curve (AUC), precision, F-score, and G-mean. These metrics were measured to ensure the effectiveness of the model.

## 6. Experimental Results

The following sections show the evaluation of the developed results using the complete dataset and the grouped dataset based on race, gender, and age.

### 6.1. Results with All Data

The experiment was conducted in eight phases. In the first phase, we analyzed the performance results of different classification algorithms for the complete dataset. Accordingly, the DT, LDA, LR, NB, SVM, kNN, Ensemble, optimized kNN, and optimized SVM algorithms provided the best results in this analysis. Thus, only the results of these classifiers are reported, as shown in Table 8. As illustrated, a different result was perceived by each classification algorithm. Compared with the other classifiers, the optimized classifiers (SVM* and kNN*) retained the highest accuracies of 0.7226 and 0.7409, respectively. Our findings suggest that the optimized classifiers achieved the best performance in the sleep apnea classification.

The kNN* offered the best result with an accuracy of (0.7409), a TPR of (0.8322), an AUC of (0.7321), a precision of (0.7294), an F-score of (0.7774), and anG-mean of (0.7252). Moreover, the kNN* achieved the highest mean rank of 1.14, thus suggesting that the kNN* was the best classifier when the complete dataset was used.

### 6.2. Data Grouping with Race

In the second phase, we inspected the performance of different classification algorithms based on the grouped data by race. Table 9 and Table 10 demonstrate the performance of different classification algorithms based on the data of Caucasian and Hispanic races, respectively. From Table 9, the highest accuracy of 0.7483 was obtained by the CKNN and kNN*. In terms of the AUC value, the KNN* retained the best AUC of 0.7114, which showed better performance in discriminating between the classes. Moreover, the kNN* yielded the optimal mean rank of 2.29. When observing the results in Table 10, it is clear that the kNN* scored the highest accuracy, TPR, TNR, AUC, precision, F-score, and G-mean. The kNN* proved to be the best algorithm in this analysis. The results of the mean rank in both Table 9 and Table 10 support this argument.

### 6.3. Data Grouping with Gender

The behavior of the different classification methods regarding the grouped data by gender is studied in this subsection. Table 11 and Table 12 outline the evaluation results of different classification algorithms. According to findings in Table 11, it is seen that the best accuracy of 0.7458 was achieved by the WKNN and Ensemble classifiers. However, the Ensemble classifier offered the optimal mean rank of 2.43, which showed excellent results for the grouped data of females. By inspecting the results in Table 12, we can observe that the performance of the kNN* was the best. The kNN* ranked first (mean rank = 1.14) and offered the highest accuracy of 0.6987, the highest TNR of 0.7500, the best AUC of 0.6875, a precision of 0.6349, an F-score of 0.6299, and a G-mean of 0.6847.

### 6.4. Data Grouping with Age

In the fourth phase, we investigated the performance of the different classification algorithms based on the grouped data by age (age ≤ 50 or age > 50). Note that the age was normally distributed around 50. Table 13 shows the evaluation results of age ≤ 50. As can be seen, the SVM* obtained the highest accuracy of 0.7523, followed by the kNN* (0.7431). Correspondingly, the SVM* contributed to the optimal TNR, AUC, precision, and G-mean. On the other side, the evaluation results of age > 50 are tabulated in Table 14. As shown, the kNN* achieved the best accuracy of 0.7333. In addition, the kNN* ranked first with the highest properties of the AUC, precision, F-score, and G-mean. Our findings indicate that the algorithms with hyperparameter optimization (SVM* and kNN*) achieved the best performance in the sleep apnea classification.

**Figure 6:**
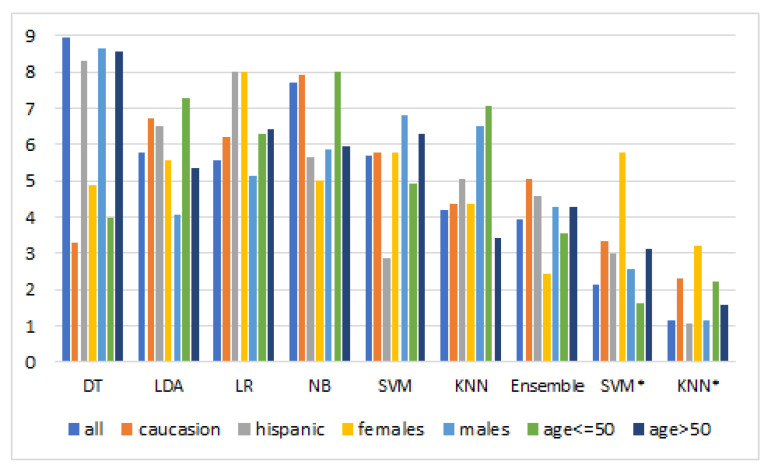
Overall ranking of classifiers. The figure displays the overall ranking of tested classifiers (DT, LDA, LR, NB, SVM, KNN, Ensemble, optimized SVM, and optimized KNN) based on the Friedman test. The rankings provide insights into the comparative performance of these classifiers, thus aiding in the identification of the most effective ones for the task at hand.

Table 15 summarizes the overall ranking results for all classifiers. Meanwhile, the bar chart of the overall ranking is demonstrated in Figure 6. As illustrated in the results, the SVM* and kNN* offered the best ranking in most cases. Among the classifiers, the SVM* and kNN* assured the optimal average rank of 3.09 and 1.80, respectively. The experimental results reveal the supremacy of the optimized algorithms for the classification of sleep apnea. The observed improvement in the kNN* and SVM* is attributed to the training process’s hyperparameter optimization, which enabled the models to explain the target concepts better.

### 6.5. Summary Performance with Data Grouping

In the fifth part of the experiment, we studied the impact of grouping (race, gender, and age) on the performance of different classifiers. Table 16 depicts the performance evaluation before and after grouping. One can see that the performances of the classifiers were substantially improved when the grouping was implemented, especially for the data grouped by races (Caucasian and Hispanic).

From the analysis, it can be inferred that the grouping step is beneficial for performance improvement. As observed in the result, the data grouped by Caucasian was the best model for accurate sleep apnea classification, with an optimal mean rank of 1.33. Based on the findings, we can conclude that grouping the data with race (Caucasian) maximizes the features’ separability between classes. Furthermore, Table 17 reports the result of the running time (in seconds). Across all the datasets, it is seen that the fastest algorithm was the DT (rank of 1.29), followed by the LDA (rank of 2.14).

## 7. Feature Selection

In the sixth phase, we investigated the impact of feature selection techniques for all cases. Generally speaking, data dimensionality has a large impact in the machine learning development process. Data with high dimensionality not only contain irrelevant and redundant features that can negatively affect the accuracy, but also require massive time and computational resources [75]. Hence, feature selection can be an effective way to resolve the above issue while improving the performance of the learning model. In this research, we adopted the most popular feature selection method, called binary particle swarm optimization (BPSO), to assess the significant features from the high-dimensional feature space. It is worth noting that the kNN* was employed as the learning algorithm, since it obtained the best performance from the previous analysis.

### 7.1. Evaluation of BPSO Using Different TFs

Initially, the BPSO with different S-shaped transfer functions (TFs) was studied. Generally, TFs play an essential role in converting the solution into a binary form. In other words, it enables the particles to search around the binary feature space. However, different TFs may yield different kinds of results [71]. Thus, we evaluated the BPSO with four other TFs and found the optimal one.

Table 18 shows the average accuracy of the BPSO variants. Based on the result obtained, the BPSO1 achieved the highest accuracy for all five cases except the complete dataset. By observing the result in Table 19, the BPSO1 also yielded the smallest number of selected features in most cases. Our results imply that the BPSO1 was highly capable of finding the optimal feature subset, thereby enhancing the learning model’s performance for sleep apnea classification. The results of the mean ranks support this clarification reported in Table 18 and Table 19.

Table 20 tabulates the running time (in seconds) of the BSPO variants. As can be observed, the BPSO1 often ran faster to find the near-optimal solution, while the BPSO2 was the slowest. Eventually, the BPSO1 was shown to be the best variant, and it was employed in the rest of the experiment.

### 7.2. Comparison of BPSO with Well-Known Algorithms

In this subsection, the performance of the BPSO1 was further compared with the other seven state-of-the-art methods. The comparison algorithms are the binary Harris hawk optimization (BHHO) [73], the binary gravitational search algorithm (BGSA) [76], the binary whale optimization algorithm (BWOA) [77], the binary grey wolf optimization (BGWO) [78], the binary bat algorithm (BBA) [79], the binary ant lion optimizer (BALO) [78], and the binary moth–flame optimization (BMFO) [48]. Table 21 presents the average accuracy results obtained by the eight different algorithms. From Table 21, it is seen that the BPSO1 outperformed the other methods in tackling the feature selection problem. The results show that the BPSO1 retained the optimal mean rank of 1.57, followed by the BHHO (2.29). Among the groups (race, gender, and age), age positively impacted accuracy when applying the BPSO1. The results revealed using feature selection showed that the performance of the grouped data by age could be substantially improved. Moreover, Table 22 presents the result of the Wilcoxon signed rank test. From Table 22, the BPSO1 outperformed the other methods in this work.

Table 23 tabulates the evaluation of the average feature size. The result of the Wilcoxon test is shown in Table 24. Our result indicates that the best algorithm in the feature reduction was the BBA, while the BSPO1 ranked second. In terms of the computational complexity, one can see from Table 25 that the BPSO1 again scored the optimal mean rank of 1.86 across all datasets. The BPSO1 offers not only the highest accuracy and the minimal number of features, but also the fastest computational speed.

**Figure 7:**
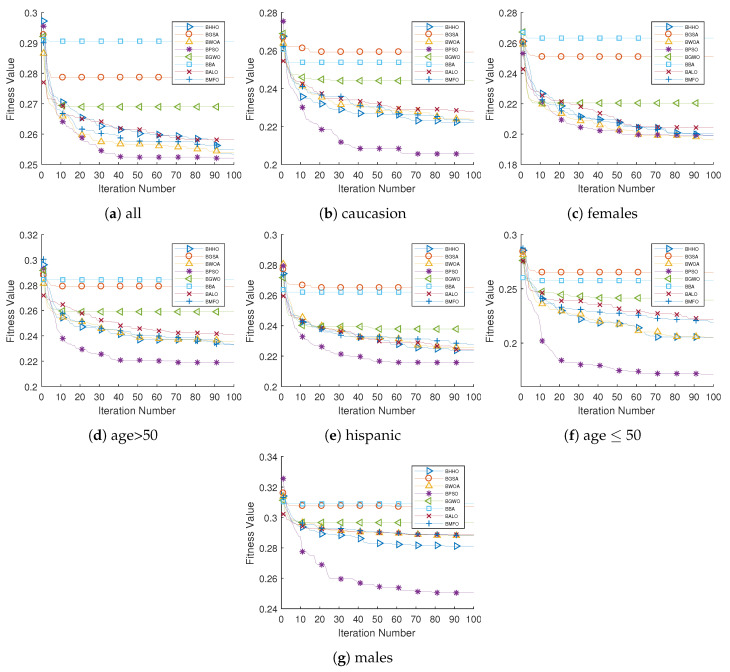
Convergence behavior of compared algorithms. The figure depicts the convergence behavior of feature selection algorithms, thereby showcasing their fitness progress over iterations and assisting in the identification of an effective approach.

Figure 7 illustrates the convergence behavior of the compared algorithms. We can observe that the BPSO1 converged faster and deeper to reach the global optimum out of all seven cases. The BPSO1 showed an excellent convergence rate against its competitors. This can be interpreted due to the strong searching ability of the BSPO1 algorithm. On the other side, the BBA and BGSA were found to have the lowest performance. They suffered from early stagnation and premature convergence, thereby reducing the classification performance.

### 7.3. Relevant Features Selected by BPSO

In the seventh phase, we inspected the relevant features selected by the BPSO1 algorithm. Table 26 outlines the best accuracy results of the classifiers with and without the BPSO algorithm. As shown in Table 26, the classification accuracy increased when the BSPO was deployed. The result affirms the importance and effectiveness of the feature selection method in sleep apnea classification. Taking the Caucasian dataset as an example, an increment of roughly 6% accuracy was achieved by the BPSO1 algorithm, with a feature reduction of 56.67%. In the dataset with age ≤ 50, the proposed approach improved the accuracy by at least 11% while eliminating more than half of the irrelevant and redundant features in the dataset. Moreover, the reduction in the feature size contributed to the overall decrease in classifier complexity.

**Figure 8:**
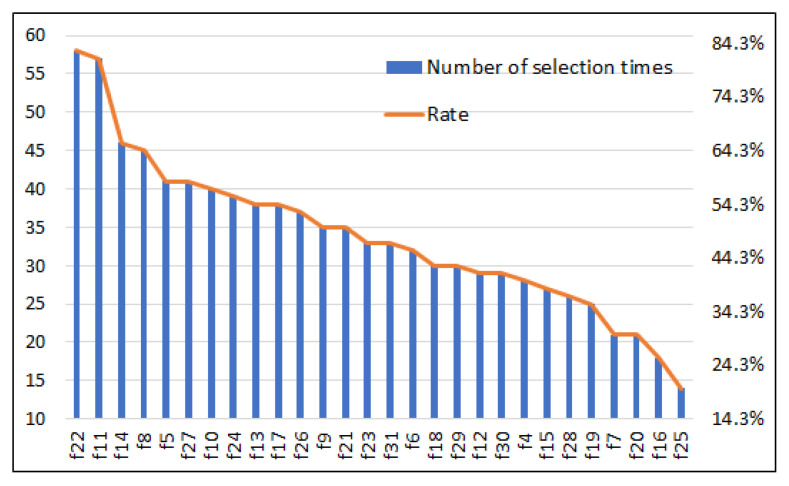
Importance of features in terms of the number of times each feature was selected by BPSO for all datasets [over 10 runs for each dataset].

Table 27 presents the details of the selected features yielded through the BPSO algorithm. Instead of using all 31 features, the results show that the number of features chosen was 18 for all datasets, 13 for the Caucasian dataset, 14 for the Hispanic dataset, 13 for the females dataset, 11 for the males dataset, 15 for the age ≤ 50 datasets, and 17 for the age > 50 datasets. The findings suggest that fewer than 20 features are sufficient for accurate sleep apnea classification. On the one hand, Figure 8 exhibits the importance of the features in terms of the number of times each feature was chosen by the BPSO. Across all the datastes, it is suggested that the most selected features were f22 and f11, followed by f14 and f8. Correspondingly, these features had high discriminative power that could best describe the OSA compared to others.

### 7.4. Comparison of the BPSO-kNN with CNN, MLP, and kNN*

In the final part of the experiments, we compared the performance of the BPSO-kNN to the kNN* and the other well-known models, including the convolutional neural network (CNN) and multilayer perceptron neural network (MLP). Note that the maximum number of epochs for both the CNN and MLP were set at 150. Table 28 presents the accuracy and computational time of the BPSO-kNN, CNN, MLP, and kNN* methods. Upon inspecting the result, the BPSO-kNN contributed to the highest accuracy for all the datasets. Although the computational complexity of the BPSO-kNN was much higher than the CNN, MLP, and kNN*, it can usually ensure an accurate classification process. All in all, our findings affirm the superiority of the BSPO-kNN for the sleep apnea classification.

**Figure 9:**
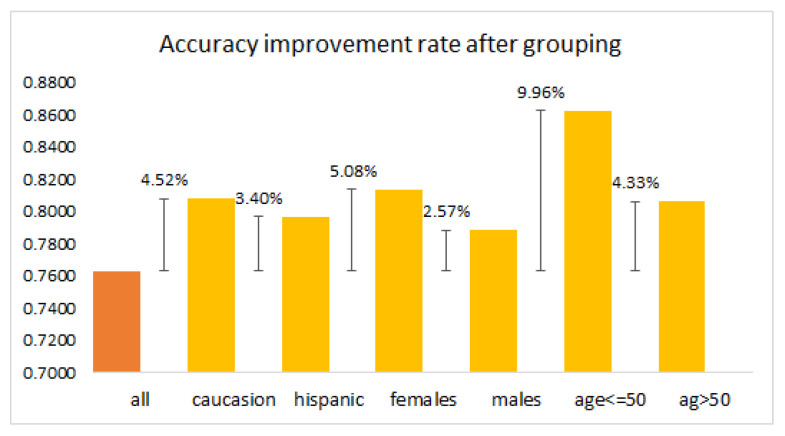
Bar chart of accuracy percentage change after grouping based on age, gender, and race. The figure compares the accuracy of the model when using the entire dataset (all) to the accuracy after grouping based on age, gender, and race. Positive values denote an increase in accuracy, thus showcasing the effectiveness of grouping based on these demographic factors.

Based on previous analysis, it showed that the performance of the OSA diagnosis can be enhanced after applying the feature selection method. According to Figure 9, the accuracy percentage showed an increment of at least 3% in most datasets. As can be observed, an increment of roughly 10% could be achieved with the feature selection approach for the dataset age ≤ 50. From the aforementioned, the irrelevant and redundant features are meaningless, and they will degrade the performance of the model, as well as increase the dimensionality of the dataset. By utilizing the BPSO-kNN, most of the unwanted features can be removed while keeping the most informative ones, which guarantees a better diagnosis of the OSA. As a bonus, the BPSO-kNN selects the useful features from the dataset in an automatic way, which means it can be implemented without the need for prior knowledge and experience. In short, feature selection is an essential and efficienct tool for sleep apnea classification.

### 7.5. Comparison Study

To verify the performance of the proposed approach, we compared the obtained results with those reported in the preceding work on the same dataset. For this purpose, the proposed BPSO-kNN was compared with the screening tool (NAMES assessment) offered by Subramanian et al. [30]. Table 29 presents the AUC scores of the NAMES assessment using different combinations of features versus the BPSO-kNN. According to the findings, it is observed that the developed BPSO-kNN outperformed the other methods, with an optimal AUC rate of 0.8320. By comparing our proposed model to [27,28], it is clear that the proposed model overwhelmed the SVM, LR, and ANN models. The results again validate the superiority of the feature selection process. These observations confirm that data grouping and the proper selection of features with an effective classification method can yield better performance for OSA detection.

## 8. Conclusions and Future Works

This study proposed an alternative approach to detect obstructive sleep apnea (OSA), which utilized demographic data instead of traditional ECG analysis. Expert physicians and sleep specialists collected a dataset of 31 features from 620 patients at the Torr Sleep Center in Texas, USA. The research focused on evaluating the performance of various machine learning classifiers using fixed and adaptive learning methods, thereby aiming to identify the most suitable classifier for the collected data. The results demonstrated that the kNN classifier achieved the highest accuracy among the tested classifiers. Additionally, a wrapper feature selection method based on the BPSO (binary particle swarm optimization) was employed with the kNN classifier to determine the most relevant features associated with OSA. The experimental outcomes indicate that the proposed method enhanced the overall prediction performance for OSA. As part of future work, the investigation will expand to include several wrapper feature selection methods, such as binary genetic algorithms (BGA) and binary ant colony optimization (BACO), thus aiming to assess the performance of the kNN classifier with different feature selection techniques. 

## Figures and Tables

**Table 1:** List of dataset features.

Attributes		Data Type
f1	Race	Categorical
f2	Age	Numeric
f3	Sex	Categorical
f4	BMI	Categorical
f5	Epworth	Numeric
f6	Wast	Numeric
f7	Hip	Numeric
f8	RDI	Numeric
f9	Neck	Numeric
f10	M.Friedman	Numeric
f11	Co-morbid	Categorical
f12	Snoring	Categorical
f13	Daytime sleepiness	Categorical
f14	DM	Categorical
f15	HTN	Categorical
f16	CAD	Categorical
f17	CVA	Categorical
f18	TST	Numeric
f19	Sleep Effic	Numeric
f20	REM AHI	Numeric
f21	NREM AHI	Numeric
f22	Supine AHI	Numeric
f23	Apnea Index	Numeric
f24	Hypopnea Index	Numeric
f25	Berlin Q	Categorical
f26	Arousal index	Numeric
f27	Awakening Index	Numeric
f28	PLM Index	Numeric
f29	Mins. SaO${}_{2}$	Numeric
f30	Mins. SaO${}_{2}$ Desats	Numeric
f31	Lowest SaO${}_{2}$	Numeric
class	Witnessed apnea	Categorical

**Table 2:** Epworth scale range.

Range	Description
0–5	Lower normal daytime sleepiness
6–10	Higher normal daytime sleepiness
11–12	Mild level of sleepiness experienced during the daytime
13–15	Moderate level of sleepiness experienced during the daytime
16–24	Significant level of sleepiness experienced during the daytime

**Table 3:** Description of sleep apnea dataset. The table provides essential information about the sleep apnea dataset, including the total number of samples (No. samples), the number of features (No. features), the count of positive samples (No.positive samples), and the count of negative samples (No. negative samples).

Datasets		No. Features	No. Samples	Negative	Positive
Original Dataset		31	274	149	125
Race	Caucasian	30	151	92	59
	Hispanic	30	123	57	66
Gender	Females	30	118	85	33
	Males	30	156	64	92
Age	Age ≤ 50	31	109	55	54
	Age > 50	31	165	94	71

**Table 4:** S-shaped transfer functions. The table provides the names and formulas of four S-shaped functions (S1, S2, S3, and S4). These functions exhibit the characteristic sigmoidal shape, which is commonly observed in S-shaped curves.

Name	Transfer Function Formula
S1	$S\left(x\right)=\frac{1}{1+e^{-2x}}$
S2	$S\left(x\right)=\frac{1}{1+e^{-x}}$
S3	$S\left(x\right)=\frac{1}{1+e^{\left(-x/2\right)}}$
S4	$S\left(x\right)=\frac{1}{1+e^{\left(-x/3\right)}}$

**Table 5:** The detailed parameter settings of preset classifiers.

Preset Classifier	Description	Parameter	Value
FDT	Fine Decision Tree	Maximum number of splits	100
		Split criterion	Gini’s diversity index
CDT	Coarse Decision Tree	Maximum number of splits	100
		Split criterion	Gini’s diversity index
LDA	Linear Discrimenant Analaysis	Discriminant type	linear
LR	Logistic Regression	-	-
GNB	Gaussian Naïve Bayes	Distribution name	Gaussian
KNB	Kernel Naïve Bayes	Distribution name	Kernel
		Kernel type	Gaussian
LSVM	Linear Support Vector Machine	Kernel function	Linear
		Kernel scale	Automatic
		Box contraint level	1
		standardize data	TRUE
MGSVM	Medium Gaussian SVM	Kernel function	Gaussian
		Kernel scale	5.6
		Box contraint level	1
		Standardize data	TRUE
CGSVM	Coarse Gaussian SVM	Kernel function	Gaussian
		Kernel scale	22
		Box contraint level	1
		Standardize data	TRUE
CKNN	Cosine K-Nearest Neighbor	Number of neighbors	10
		Distance metric	cosine
		Distance weight	equal
		Standardize data	TRUE
WKNN	Weighted kNN	Number of neighbors	10
		Distance metric	Euclidean
		Distance weight	Squared Inverse
		Standardize data	TRUE
Ensemble	Subspace Discriminant	Ensemble method	Subspace
		Learner type	Discriminant
		Number of learners	30
		Subspace dimension	16

**Table 6:** Parameter settings of the optimized kNN for each dataset.

Dataset	Number of Neighbors	Distance Metric	Distance Weight	Standardize Data
All	16	Spearman	Inverse	TRUE
Caucasian	6	Correlation	Squared Inverse	TRUE
Hispanic	32	Cityblock	Squared Inverse	TRUE
Females	4	Hamming	Squared Inverse	FALSE
Males	22	Cityblock	Equal	FALSE
Age ≤ 50	14	Cosine	Squared Inverse	TRUE
Age > 50	31	16	Squared Inverse	TRUE

**Table 7:** Parameter settings of the optimized SVM for each dataset.

Dataset	Kernel Function	Kernel Scale	Box Contraint Level	Standardized Data
All	Polynomial (degree = 2)	1	0.002351927	TRUE
Caucasian	Linear	1	0.18078	TRUE
Hispanic	Linear	1	0.01115743	TRUE
Females	Gaussian	2.990548535	122.3491994	FALSE
Males	Linear	1	0.001000015	FALSE
Age ≤ 50	Gaussian	415.5625146	341.7329909	FALSE
Age > 50	Gaussian	26.42211158	7.503025335	TRUE

**Table 8:** Performance of different classification algorithms on the data, where X* denotes the optimized classifier X.

Classifier	Accuracy	TPR	TNR	AUC	Precision	F-Score	G-Mean	Mean Rank
DT	0.5876	0.6309	0.5360	0.5834	0.6184	0.6246	0.5815	8.97
LDA	0.6861	0.7584	0.6000	0.6792	0.6933	0.7244	0.6746	5.79
LR	0.6861	0.7383	0.6240	0.6811	0.7006	0.7190	0.6787	5.57
NB	0.6642	0.7785	0.5280	0.6533	0.6629	0.7160	0.6411	7.71
SVM	0.6898	0.8188	0.5360	0.6774	0.6778	0.7416	0.6625	5.71
kNN	0.6934	0.7517	0.6240	0.6878	0.7044	0.7273	0.6849	4.21
Ensemble	0.7044	0.8188	0.5680	0.6934	0.6932	0.7508	0.6820	3.93
SVM*	0.7226	0.7919	0.6400	0.7160	0.7239	0.7564	0.7119	2.14
kNN*	**0.7409**	**0.8322**	0.6320	**0.7321**	**0.7294**	**0.7774**	**0.7252**	**1.14**

**Table 9:** Performance of different classification algorithms for the grouped data of Caucasian race.

Classifier	Accuracy	TPR	TNR	AUC	Precision	F-Score	G-Mean	Mean Rank
FDT	0.7285	0.7935	**0.6271**	0.7103	**0.7684**	0.7807	**0.7054**	3.29
LDA	0.6908	0.7957	0.5254	0.6606	0.7255	0.7590	0.6466	6.71
LR	0.6887	0.7609	0.5763	0.6686	0.7368	0.7487	0.6622	6.21
GNB	0.6887	0.8261	0.4746	0.6503	0.7103	0.7638	0.6261	7.93
MGSVM	0.7219	0.8913	0.4576	0.6745	0.7193	0.7961	0.6387	5.79
CKNN	**0.7483**	**0.9457**	0.4407	0.6932	0.7250	**0.8208**	0.6455	4.36
Ensemble	0.7219	0.8696	0.4915	0.6805	0.7273	0.7921	0.6538	5.07
SVM*	0.7351	0.8478	0.5593	0.7036	0.7500	0.7959	0.6886	3.36
kNN*	**0.7483**	0.8804	0.5424	**0.7114**	0.7500	0.8100	0.6910	**2.29**

**Table 10:** Performance of different classification algorithms for the grouped data of Hispanic race.

Data	Accuracy	TPR	TNR	AUC	Precision	F-Score	G-Mean	Mean Rank
CDT	0.6260	0.5263	0.7121	0.6192	0.6122	0.5660	0.6122	8.29
LDA	0.6423	0.5614	0.7121	0.6368	0.6275	0.5926	0.6323	6.50
LR	0.6260	0.5614	0.6818	0.6216	0.6038	0.5818	0.6187	8.00
GNB	0.6504	0.6140	0.6818	0.6479	0.6250	0.6195	0.6470	5.64
MGSVM	0.6829	0.5614	0.7879	0.6746	0.6957	0.6214	0.6651	2.86
WKNN	0.6585	0.5439	0.7576	0.6507	0.6596	0.5962	0.6419	5.07
Ensemble	0.6585	0.5965	0.7121	0.6543	0.6415	0.6182	0.6517	4.57
SVM*	0.6748	**0.6316**	0.7121	0.6719	0.6545	0.6429	0.6706	3.00
kNN*	**0.7724**	**0.6316**	**0.8939**	**0.7628**	**0.8372**	**0.7200**	**0.7514**	**1.07**

**Table 11:** Performance of different classification algorithms for the grouped data of females.

Classifier	Accuracy	TPR	TNR	AUC	Precision	F-Score	G-Mean	Mean Rank
CDT	0.7119	0.8824	0.2727	0.5775	0.7576	0.8152	0.4906	4.86
LDA	0.6695	0.8118	0.3030	0.5574	0.7500	0.7797	0.4960	5.57
LR	0.6102	0.7529	0.2424	0.4977	0.7191	0.7356	0.4272	8.00
KNB	0.6356	0.7176	**0.4242**	0.5709	0.7625	0.7394	0.5518	5.00
MGSVM	0.7203	**1.0000**	0.0000	0.5000	0.7203	0.8374	0.0000	5.79
WKNN	**0.7458**	0.9765	0.1515	0.5640	0.7477	**0.8469**	0.3846	4.36
Ensemble	**0.7458**	0.8824	0.3939	**0.6381**	**0.7895**	0.8333	**0.5896**	**2.43**
SVM*	0.7203	**1.0000**	0.0000	0.5000	0.7203	0.8374	0.0000	5.79
kNN*	0.7373	0.9176	0.2727	0.5952	0.7647	0.8342	0.5003	3.21

**Table 12:** Performance of different classification algorithms for the grouped data of males.

Classifier	Accuracy	TPR	TNR	AUC	Precision	F-Score	G-Mean	Mean Rank
CDT	0.5256	0.2969	0.6848	0.4908	0.3958	0.3393	0.4509	8.64
LDA	0.6538	0.5938	0.6957	0.6447	0.5758	0.5846	0.6427	4.07
LR	0.6474	0.5938	0.6848	0.6393	0.5672	0.5802	0.6376	5.14
KNB	0.6234	**0.6406**	0.6111	0.6259	0.5395	0.5857	0.6257	5.86
LSVM	0.6346	0.5313	0.7065	0.6189	0.5574	0.5440	0.6126	6.79
CKNN	0.6282	0.6094	0.6413	0.6253	0.5417	0.5735	0.6251	6.50
Ensemble	0.6603	0.5469	0.7391	0.6430	0.5932	0.5691	0.6358	4.29
SVM*	0.6667	0.6094	0.7065	0.6579	0.5909	0.6000	0.6562	2.57
kNN*	**0.6987**	0.6250	**0.7500**	**0.6875**	**0.6349**	**0.6299**	**0.6847**	**1.14**

**Table 13:** Performance of different classification algorithms for the grouped data by age (Age ≤ 50).

Classifier	Accuracy	TPR	TNR	AUC	Precision	F-Score	G-Mean	Mean Rank
FDT	0.7064	0.7091	0.7037	0.7064	0.7091	0.7091	0.7064	4.00
LDA	0.6514	0.6727	0.6296	0.6512	0.6491	0.6607	0.6508	7.29
LR	0.6697	0.7091	0.6296	0.6694	0.6610	0.6842	0.6682	6.29
KNB	0.6239	0.5636	0.6852	0.6244	0.6458	0.6019	0.6214	8.00
LSVM	0.7064	0.8000	0.6111	0.7056	0.6769	0.7333	0.6992	4.93
CKNN	0.6514	0.8182	0.4815	0.6498	0.6164	0.7031	0.6276	7.07
Ensemble	0.7156	0.7818	0.6481	0.7150	0.6935	0.7350	0.7119	3.57
SVM*	**0.7523**	0.7818	**0.7222**	**0.7520**	**0.7414**	0.7611	**0.7514**	**1.64**
kNN*	0.7431	**0.8364**	0.6481	0.7423	0.7077	**0.7667**	0.7363	2.21

**Table 14:** Performance of different classification algorithms for the grouped data by age (Age > 50).

Classifier	Accuracy	TPR	TNR	AUC	Precision	F-Score	G-Mean	Mean Rank
CDT	0.6061	0.6702	0.5211	0.5957	0.6495	0.6597	0.5910	8.57
LDA	0.6667	0.7234	**0.5915**	0.6575	0.7010	0.7120	0.6542	5.36
LR	0.6606	0.7234	0.5775	0.6504	0.6939	0.7083	0.6463	6.43
KNB	0.6788	0.7979	0.5211	0.6595	0.6881	0.7389	0.6448	5.93
CGSVM	0.6727	**0.9149**	0.3521	0.6335	0.6515	0.7611	0.5676	6.29
CKNN	0.6909	0.7766	0.5775	0.6770	0.7087	0.7411	0.6697	3.43
Ensemble	0.6909	0.7979	0.5493	0.6736	0.7009	0.7463	0.6620	4.29
SVM*	0.7091	0.8511	0.5211	0.6861	0.7018	0.7692	0.6660	3.14
KNN*	**0.7333**	0.8617	0.5634	**0.7125**	**0.7232**	**0.7864**	**0.6968**	**1.57**

**Table 15:** Overall ranking results for all classifiers in dealing with different datasets based on classification evaluation metrics reported in Table 5, Table 6, Table 7, Table 8, Table 9, Table 10 and Table 11.

Classifier	All	Caucasian	Hispanic	Females	Males	Age ≤ 50	Age > 50	Average Rank
DT	8.97	3.29	8.29	4.86	8.64	4.00	8.57	6.66
LDA	5.79	6.71	6.50	5.57	4.07	7.29	5.36	5.90
LR	5.57	6.21	8.00	8.00	5.14	6.29	6.43	6.52
NB	7.71	7.93	5.64	5.00	5.86	8.00	5.93	6.58
SVM	5.71	5.79	2.86	5.79	6.79	4.93	6.29	5.45
KNN	4.21	4.36	5.07	4.36	6.50	7.07	3.43	5.00
Ensemble	3.93	5.07	4.57	**2.43**	4.29	3.57	4.29	4.02
SVM*	2.14	3.36	3.00	5.79	2.57	**1.64**	3.14	3.09
KNN*	**1.14**	**2.29**	**1.07**	3.21	**1.14**	2.21	**1.57**	**1.80**

**Table 16:** Performance evaluation before and after grouping in terms of accuracy measure.

Classifier	All	Caucasian	Hispanic	Females	Males	Age ≤ 50	Age > 50
DT	0.5876	**0.7285**	0.6260	0.7119	0.5256	0.7064	0.6061
LDA	0.6861	**0.6908**	0.6423	0.6695	0.6538	0.6514	0.6667
LR	0.6861	**0.6887**	0.6260	0.6102	0.6474	0.6697	0.6606
NB	0.6642	**0.6887**	0.6504	0.6356	0.6234	0.6239	0.6788
SVM	0.6898	**0.7219**	0.6829	0.7203	0.6346	0.7064	0.6727
kNN	0.6934	**0.7483**	0.6585	0.7458	0.6282	0.6514	0.6909
Ensemble	0.7044	0.7219	0.6585	**0.7458**	0.6603	0.7156	0.6909
SVM*	0.7226	0.7351	0.6748	0.7203	0.6667	**0.7523**	0.7091
kNN*	0.7409	0.7483	**0.7724**	0.7373	0.6987	0.7431	0.7333
mean Rank	3.44	**1.33**	5.00	3.44	6.44	3.78	4.56

**Table 17:** Comparison between classification algorithms dealing with all datasets in terms of running time in seconds.

Classifier	All	Caucasion	Hispanic	Females	Males	Age ≤ 50	Age > 50	Average Rank
DT	1.7018	0.1440	0.1093	0.1185	0.1123	0.0911	0.1280	1.29
LDA	1.6479	0.1742	0.1670	0.1720	0.1729	0.1400	0.1654	2.14
LR	0.3802	0.3067	0.2393	0.2837	0.2654	0.2357	0.2433	4.00
NB	3.6635	2.1350	3.0485	2.7358	3.8645	4.1244	3.2748	6.86
SVM	2.9095	0.2939	0.2926	0.2804	0.2520	0.2577	0.2710	4.57
KNN	1.7064	0.1768	0.1940	0.1648	0.2247	0.1761	0.1751	3.00
Ensemble	4.2913	1.9599	2.1277	1.8031	1.9468	2.0706	2.0714	6.14

**Table 18:** Comparison between different BPSO variants using four S-shaped TFs in terms of average accuracy based on kNN* classifier.

Dataset	Measure	BPSO1	BPSO2	BPSO3	BPSO4
All	AVG	0.7511	**0.7518**	0.7464	0.7449
	STD	0.0075	0.0045	0.0039	0.0040
Caucasian	AVG	**0.7967**	0.7954	0.7841	0.7808
	STD	0.0136	0.0114	0.0084	0.0091
Females	AVG	**0.8034**	0.7941	0.7949	0.7907
	STD	0.0078	0.0070	0.0067	0.0098
Age > 50	AVG	**0.7836**	0.7782	0.7752	0.7655
	STD	0.0111	0.0111	0.0092	0.0064
Hispanic	AVG	**0.7870**	0.7862	0.7797	0.7740
	STD	0.0064	0.0067	0.0071	0.0107
Age ≤ 50	AVG	**0.8321**	0.8211	0.8092	0.7945
	STD	0.0168	0.0099	0.0149	0.0131
Males	AVG	**0.7513**	0.7205	0.7186	0.7180
	STD	0.0159	0.0081	0.0047	0.0043
Mean Rank	F-test	**1.14**	2.00	2.86	4.00

**Table 19:** Comparison between different BPSO variants using four S-shaped TFs in terms of average number of selected features [based on kNN*].

Dataset	Measure	BPSO1	BPSO2	BPSO3	BPSO4
All	AVG	17.6	**16.4**	17.7	16.9
	STD	2.5473	3.0984	2.8304	2.6854
Caucasian	AVG	**13.0**	13.7	14.9	14.5
	STD	3.2660	2.9458	3.4785	2.7988
Females	AVG	14.1	14.4	14.8	**13.2**
	STD	1.8529	2.2211	2.0440	3.1903
Age > 50	AVG	**14.8**	15.0	15.4	16.0
	STD	2.6583	2.4944	1.8974	3.4641
Hispanic	AVG	**14.6**	14.7	15.4	16.2
	STD	0.6992	2.0575	2.5473	2.8206
Age ≤ 50	AVG	**14.8**	15.6	15.4	15.5
	STD	1.3166	1.7764	2.3190	2.5927
Males	AVG	**12.5**	12.8	14.8	13.6
	STD	2.8771	3.1903	2.4404	1.3499
Mean Rank	F-test	**1.43**	2.29	3.43	2.86

**Table 20:** Comparison between different BPSO variants using four S-shaped TFs in terms of average running time [based on kNN*].

Dataset	Measure	BPSO1	BPSO2	BPSO3	BPSO4
All	AVG	**464.0**	481.7	466.2	467.8
	STD	4.8617	3.6674	3.3655	3.2812
caucasion	AVG	**368.1**	374.5	371.8	374.0
	STD	4.3402	4.2569	3.6641	3.5733
females	AVG	**245.8**	247.9	247.9	248.4
	STD	2.1698	2.1417	1.7136	2.1340
age > 50	AVG	**266.9**	270.2	268.7	270.0
	STD	2.5395	2.2341	2.4543	1.5417
hispanic	AVG	**260.5**	262.9	262.1	263.9
	STD	2.9500	2.1613	1.8231	2.4382
age ≤ 50	AVG	**261.4**	262.1	264.8	263.7
	STD	2.3115	1.7837	2.1478	2.0247
males	AVG	382.7	251.8	**250.3**	250.9
	STD	46.5774	1.8658	1.8342	1.7393
mean rank	F-test	**1.43**	3.21	2.21	3.14

**Table 21:** Comparison between BPSO and various well-know algorithms in terms of average accuracy.

Dataset	Measure	BPSO1	BHHO	BGSA	BWOA	BGWO	BBA	BALO	BMFO
All	AVG	0.7511	**0.7515**	0.7245	0.7507	0.7372	0.6624	0.7474	0.7504
	STD	0.0075	0.0095	0.0086	0.0042	0.0064	0.0472	0.0038	0.0039
Caucasian	AVG	**0.7967**	0.7821	0.7430	0.7815	0.7623	0.7060	0.7781	0.7821
	STD	0.0136	0.0049	0.0061	0.0054	0.0049	0.0356	0.0056	0.0058
Females	AVG	0.8034	0.8068	0.7517	**0.8093**	0.7864	0.6949	0.8017	0.8059
	STD	0.0078	0.0067	0.0106	0.0072	0.0088	0.0344	0.0091	0.0063
Age > 50	AVG	**0.7836**	0.7703	0.7236	0.7691	0.7473	0.6945	0.7649	0.7721
	STD	0.0111	0.0078	0.0122	0.0097	0.0081	0.0192	0.0110	0.0077
Hispanic	AVG	**0.7870**	0.7805	0.7382	0.7789	0.7683	0.6805	0.7813	0.7772
	STD	0.0064	0.0094	0.0120	0.0064	0.0103	0.0617	0.0071	0.0042
Age ≤ 50	AVG	**0.8321**	0.7991	0.7376	0.7991	0.7661	0.6661	0.7835	0.7853
	STD	0.0168	0.0110	0.0189	0.0091	0.0108	0.0604	0.0151	0.0064
Males	AVG	**0.7513**	0.7224	0.6949	0.7154	0.7090	0.6430	0.7160	0.7160
	STD	0.0159	0.0053	0.0106	0.0033	0.0033	0.0327	0.0031	0.0031
Mean Rank	F-test	**1.57**	2.29	7.00	3.36	6.00	8.00	4.36	3.43

**Table 22:** *p*-values of the Wilcoxon signed rank test based on accuracy results reported in Table 21 (*p*-values ≤ 0.05 are in bold and significant).

Dataset	BPSO (the Best Performaing Method) vs.						
	BHHO	BGSA	BWOA	BGWO	BBA	BALO	BMFO
All	$2.79\times 10^{-1}$	$2.62\times 10^{-4}$	$5.05\times 10^{-1}$	$1.54\times 10^{-3}$	$1.69\times 10^{-4}$	$4.82\times 10^{-2}$	$5.55\times 10^{-1}$
Caucasian	$8.58\times 10^{-3}$	$1.51\times 10^{-4}$	$1.12\times 10^{-2}$	$2.27\times 10^{-4}$	$1.66\times 10^{-4}$	$5.03\times 10^{-3}$	$1.13\times 10^{-2}$
Females	$3.53\times 10^{-1}$	$1.50\times 10^{-4}$	$1.16\times 10^{-1}$	$1.15\times 10^{-3}$	$1.60\times 10^{-4}$	$4.69\times 10^{-1}$	$5.13\times 10^{-1}$
Age > 50	$8.05\times 10^{-3}$	$1.74\times 10^{-4}$	$8.19\times 10^{-3}$	$1.62\times 10^{-4}$	$1.73\times 10^{-4}$	$2.93\times 10^{-3}$	$1.83\times 10^{-2}$
Hispanic	$1.04\times 10^{-1}$	$1.56\times 10^{-4}$	$1.89\times 10^{-2}$	$4.39\times 10^{-4}$	$1.62\times 10^{-4}$	$1.18\times 10^{-1}$	$2.24\times 10^{-3}$
Age ≤ 50	$3.85\times 10^{-4}$	$1.64\times 10^{-4}$	$3.28\times 10^{-4}$	$1.58\times 10^{-4}$	$1.71\times 10^{-4}$	$1.61\times 10^{-4}$	$1.45\times 10^{-4}$
Males	$9.68\times 10^{-1}$	$1.51\times 10^{-4}$	$8.81\times 10^{-3}$	$2.72\times 10^{-4}$	$1.57\times 10^{-4}$	$1.26\times 10^{-2}$	$1.26\times 10^{-2}$

**Table 23:** Comparison between BPSO and various well-know algorithms based on the number of selected features.

Dataset	Measure	BPSO1	BHHO	BGSA	BWOA	BGWO	BBA	BALO	BMFO
All	AVG	17.6	22.8	18.1	22.2	27.4	**13**	25.1	24.2
	STD	2.55	2.10	2.23	2.66	1.17	2.11	1.37	1.81
Caucasian	AVG	13.0	19.9	14.6	21.5	26.2	**12.1**	24.6	21.7
	STD	3.27	3.28	2.63	2.32	0.79	2.33	1.35	1.89
Females	AVG	14.1	23	15	22.8	27	**13.4**	24	24.8
	STD	1.85	1.56	1.89	2.39	1.56	2.17	1.33	1.69
Age > 50	AVG	**14.8**	18.6	16.9	21.3	27.3	15.1	25.6	22.5
	STD	2.66	5.87	2.88	3.47	1.64	2.42	1.65	2.12
Hispanic	AVG	14.6	19.6	17.3	20.7	25.2	**13**	23.3	21.1
	STD	0.70	3.78	3.53	3.59	1.23	2.45	1.89	2.38
Age ≤ 50	AVG	14.8	18.3	16.4	19	25.1	**12.6**	23.4	19.5
	STD	1.32	2.11	2.80	1.83	1.20	2.46	1.51	1.18
Males	AVG	12.5	19.1	14.9	18.6	25.5	**11.3**	22.5	21.4
	STD	2.88	5.24	1.60	3.75	1.84	4.57	1.96	2.27
Mean Rank	F-test	1.86	4.43	3.00	4.57	8.00	1.14	6.86	6.14

**Table 24:** *p*-values of the Wilcoxon signed rank test based on the number of selected features reported in Table 23 (*p*-values ≤ 0.05 are in bold and significant).

Dataset	BPSO (the Best Performaing Method) vs.						
	BHHO	BGSA	BWOA	BGWO	BBA	BALO	BMFO
$9.43\times 10^{-4}$	$7.01\times 10^{-1}$	$2.35\times 10^{-3}$	$1.70\times 10^{-4}$	$1.42\times 10^{-3}$	$1.73\times 10^{-4}$	$2.12\times 10^{-4}$	
Caucasian	$7.44\times 10^{-4}$	$2.37\times 10^{-1}$	$2.65\times 10^{-4}$	$1.57\times 10^{-4}$	$5.41\times 10^{-1}$	$1.63\times 10^{-4}$	$2.02\times 10^{-4}$
Females	$1.60\times 10^{-4}$	$2.30\times 10^{-1}$	$1.65\times 10^{-4}$	$1.61\times 10^{-4}$	$4.16\times 10^{-1}$	$1.62\times 10^{-4}$	$1.51\times 10^{-4}$
Age > 50	$1.02\times 10^{-1}$	$1.37\times 10^{-1}$	$7.30\times 10^{-4}$	$1.67\times 10^{-4}$	$7.31\times 10^{-1}$	$1.56\times 10^{-4}$	$1.73\times 10^{-4}$
Hispanic	$1.37\times 10^{-2}$	$1.20\times 10^{-2}$	$1.40\times 10^{-3}$	$1.43\times 10^{-4}$	$1.08\times 10^{-1}$	$1.51\times 10^{-4}$	$1.44\times 10^{-4}$
Age ≤ 50	$1.58\times 10^{-3}$	$1.62\times 10^{-1}$	$4.79\times 10^{-4}$	$1.67\times 10^{-4}$	$2.65\times 10^{-2}$	$1.70\times 10^{-4}$	$1.61\times 10^{-4}$
Males	$3.42\times 10^{-3}$	$1.82\times 10^{-2}$	$1.64\times 10^{-3}$	$1.68\times 10^{-4}$	$7.61\times 10^{-1}$	$2.00\times 10^{-4}$	$2.03\times 10^{-4}$

**Table 25:** Comparison between BPSO and various well-know algorithms in terms of running time (in seconds).

Dataset	Measure	BPSO1	BHHO	BGSA	BWOA	BGWO	BBA	BALO	BMFO
all	AVG	**464.05**	798.02	465.45	476.24	475.84	468.76	474.47	468.78
	STD	4.862	9.414	4.992	4.813	5.720	5.622	7.119	6.179
caucasion	AVG	**368.14**	613.92	376.20	378.73	377.41	376.77	375.30	374.28
	STD	4.340	5.295	3.015	3.928	4.045	3.191	5.114	4.507
females	AVG	**245.75**	401.38	248.90	248.18	249.38	250.27	247.90	247.08
	STD	2.170	4.390	2.088	2.839	2.664	1.802	2.385	2.445
age > 50	AVG	**266.88**	441.47	267.54	272.05	272.21	269.97	269.77	269.41
	STD	2.540	4.207	2.024	2.318	2.357	2.169	3.116	2.618
hispanic	AVG	**260.48**	431.94	264.26	261.83	264.53	265.01	262.17	261.13
	STD	2.950	4.529	1.983	2.527	2.019	3.110	2.884	1.943
age ≤ 50	AVG	**261.38**	429.46	266.24	263.29	266.10	265.21	262.29	262.48
	STD	2.311	3.038	2.525	1.785	2.330	2.237	3.266	1.700
males	AVG	382.68	409.66	250.95	249.72	251.26	255.21	**249.02**	249.54
	STD	46.577	4.520	1.540	2.240	1.781	3.173	2.732	2.101
mean rank	F-test	**1.86**	8.00	4.14	4.86	6.00	5.43	3.14	2.57

**Table 26:** Best results for classifiers without using feature selection (kNN*) and after using feature selection (BPSO-kNN*) in terms of accuracy, number of features, and improvement rate.

Dataset	KNN*		BPSO-KNN		Improvement Rate	
	Accuracy	No. Features	Accuracy	No. Features	Features Reduction	Accuracy
All	0.7409	31	**0.7628**	**18**	41.94%	2.19%
Caucasion	0.7483	30	**0.8080**	**13**	56.67%	5.96%
Hispanic	0.7724	30	**0.7968**	**14**	53.33%	2.44%
Females	0.7373	30	**0.8136**	**13**	56.67%	7.63%
Males	0.6987	30	**0.7885**	**11**	63.33%	8.97%
Age ≤ 50	0.7431	31	**0.8624**	**15**	51.61%	11.93%
Age > 50	0.7333	31	**0.8061**	**17**	45.16%	7.27%

**Table 27:** Details of selected features selected by the BPSO that scored the best accuracy results for each dataset [best result out of 10 runs].

Dataset	Accuracy	#Features	f1	f2	f3	f4	f5	f6	f7	f8	f9	f10	f11	f12	f13	f14	f15	f16	f17	f18	f19	f20	f21	f22	f23	f24	f25	f26	f27	f28	f29	f30	f31
all	**0.7628**	18	0	0	1	0	1	1	0	1	0	1	1	0	1	1	0	0	0	1	1	0	1	1	1	0	1	1	1	0	0	1	1
caucasian	**0.8080**	13	-	0	1	0	1	1	0	1	0	1	1	0	1	1	0	0	0	0	0	0	1	1	0	0	0	0	0	1	1	0	1
Hispanic	**0.7968**	14	-	1	0	1	0	0	0	1	1	0	1	1	0	1	0	0	1	0	0	0	1	1	0	1	0	1	0	1	0	1	0
females	**0.8136**	13	1	0	-	0	1	1	0	0	0	1	1	0	1	1	0	0	0	1	1	0	1	0	0	1	0	0	0	0	1	0	1
males	**0.7885**	11	0	0	-	0	1	0	0	0	0	1	1	0	1	0	0	0	0	0	1	0	0	1	1	1	0	1	0	0	1	0	1
age<=50	**0.8624**	15	0	0	1	0	0	1	0	1	1	0	0	0	1	1	1	0	1	0	0	1	0	1	1	1	0	1	0	0	0	1	1
age>50	**0.8061**	17	1	0	0	0	0	1	1	1	1	1	1	1	0	0	1	0	1	0	1	0	0	1	1	0	0	1	1	0	1	0	1

**Table 28:** Comparison of the BPSO-kNN with kNN*, CNN, and MLP. The table compares the performance of the proposed model, BPSO-kNN, which incorporates feature selection, with other models, including kNN*, CNN, and MLP, which do not employ feature selection.

Dataset	CNN		MLP		kNN*		BPSO-kNN	
	Accuracy	Time	Accuracy	Time	Accuracy	Time	Accuracy	Time
All	0.6105	291.656	0.5438	0.789	0.7409	1.706	**0.7628**	464.050
Caucasion	0.7283	204.591	0.6159	3.282	0.7483	0.177	**0.8080**	368.142
Hispanic	0.6513	180.510	0.5285	2.341	0.7724	0.194	**0.7968**	245.754
Females	0.7023	208.605	0.6102	3.398	0.7373	0.165	**0.8136**	266.882
Males	0.6263	174.349	0.5769	2.969	0.6987	0.225	**0.7885**	260.481
Age ≤ 50	0.6427	177.557	0.5780	2.917	0.7431	0.176	**0.8624**	261.377
Age > 50	0.6629	219.354	0.5455	3.483	0.7333	0.175	**0.8061**	382.676

**Table 29:** Comparison of the proposed BPSO-kNN with other approaches from the literature in terms of AUC scores.

Results of NAMES [30]		Proposed (BPSO-KNN)		Haberfeld et al. [28]		Surani et al. [27]	
**Combination**	**AUC**	**Dataset**	**Average AUC**	**SVM**	**LR**	**LR**	**ANN**
NC + MF + CM + ESS + S + BMI	0.6577	all	**0.7438**				
NC + MF + CM + ESS + S + M	0.6572	caucasion	**0.7690**				
NC + MF + CM + ESS + S + BMI + M (NAMES2)	0.6690	hispanic	**0.7811**				
NC + MF + M + ESS + S	0.6583	females	**0.6707**	0.6220	0.6080	0.7030	0.5830
BMI + MF + CM + ESS + S + M	0.6436	males	**0.7318**	0.6070	0.6070	0.7130	0.6360
(NC + MF) × 2 + CM + ESS + S	0.6661	age ≤ 50	**0.8320**				
(NC + BMI) × 2 + M + ESS + S	0.6433	ag > 50	**0.7684**				
(NC + MF) × 2 + M + ESS + S	0.6484						
(NC + BMI) × 2 + CM + ESS + S	0.6426						
(NC + MF + BMI)×2 + CM + ESS + S + M	0.6478						

## Data Availability

Not applicable.
